# Factors Affecting Vancomycin and Piperacillin–Tazobactam-Induced Nephrotoxicity

**DOI:** 10.3390/jcm15135020

**Published:** 2026-06-27

**Authors:** Mollie VanNatta Day, Ruhul Munshi, Kalyn Picard, Alexandre E. Malek

**Affiliations:** 1 Ochsner LSU Health Shreveport, Academic Medical Center, 1541 Kings Highway, Shreveport, LA 71103, USA; alexandre.malek@lsuhs.edu; 2 Division of Infectious Disease, Department of Medicine, School of Medicine, LSU Health Shreveport, 1501 Kings Highway, Shreveport, LA 71103, USA; ramin166@outlook.com (R.M.); kmp006@lsuhs.edu (K.P.)

**Keywords:** nephrotoxicity, vancomycin, piperacillin–tazobactam, antimicrobial stewardship

## Abstract

**Background/Objectives:** Nephrotoxicity caused by use of vancomycin and piperacillin–tazobactam has been heavily debated, and specific impact of this combination on development of acute kidney injury (AKI) has remained uncertain. We sought to further elucidate the risk factors contributing to nephrotoxicity. **Methods:** We conducted a retrospective cohort study at our academic medical center from May 2019 to March 2023. We included adult patients who received at least 48 h of dual vancomycin and piperacillin–tazobactam therapy. The primary outcome is incidence of kidney injury along with determinants of AKI. Patients were stratified into two groups according to occurrence of AKI or its absence. Demographic features, concomitant use of nephrotoxic agents, site of infection, use of vasopressors, duration of therapy, and vancomycin-level parameters were compared. **Results:** Of 350 patients, AKI occurred in 89 patients (25.4%). The 30-day mortality was comparable between the two groups. No significant differences were observed in age, gender, race, or comorbidities, although more AKI cases occurred in African American patients (51.7%). Elevated initial vancomycin trough and estimated area under the curve/minimum inhibitory concentration levels, higher mean body mass index (BMI), and use of intravenous (IV) contrast were more frequently observed in the AKI group. **Conclusions:** We demonstrated that 25.4% of patients receiving vancomycin and piperacillin–tazobactam combination experienced AKI, while higher BMI and use of concomitant IV contrast were significantly associated with increased nephrotoxicity. Therefore, we suggest proceeding with caution regarding use of vancomycin and piperacillin–tazobactam in patients with elevated BMI and those who have received IV contrast.

## 1. Introduction

Nephrotoxicity, a serious adverse effect leading to acute kidney injury (AKI), is a frequently reported concern associated with the use of vancomycin and piperacillin–tazobactam combination therapy. Vancomycin, a glycopeptide antibiotic, is commonly used to treat serious Gram-positive infections, including methicillin-resistant *Staphylococcus aureus* (MRSA), while piperacillin–tazobactam, a combination of a broad-spectrum penicillin and a beta-lactamase inhibitor, is effective against a wide array of Gram-negative, Gram-positive, and anaerobic organisms, including *Pseudomonas aeruginosa* [1,2]. The combination of these two antimicrobials is an attractive option for empiric coverage against the most common causes of severe infections that require hospitalizations; however, AKI can prolong hospitalizations, increase morbidity, decrease long-term survival rates, and increase costs.

Several factors contribute to the nephrotoxic effects of vancomycin and piperacillin–tazobactam, including pharmacokinetic and pharmacodynamic properties which play crucial roles in mediating these adverse effects. According to a meta-analysis performed by Van Hal and colleagues, the incidence of vancomycin-associated AKI across published studies has varied widely from 5% to 43% [2]. Vancomycin-induced nephrotoxicity is primarily linked to elevated serum concentration levels. Target serum levels correlate with an area under the curve (AUC)/minimum inhibitory concentration (MIC) ratio of 400 to 600 mg·h/L. This target has been extensively studied and deemed to be the appropriate range to optimize efficacy while reducing the risk of AKI. The vancomycin therapeutic monitoring guidelines identify an increased risk of AKI with AUC values between 600 and 800, specifically those greater than 650 mg·h/L [3]. Serum trough levels have been used historically as a surrogate marker of AUC/MIC, which requires complex formulas to calculate. A trough concentration of 15 to 20 mg/L is the surrogate marker for the optimal vancomycin AUC/MIC if the MIC is ≤1 mg/L in patients with normal renal function. Therefore, high trough concentrations (≥20 mg/L) have been associated with an increased risk of nephrotoxicity as they likely correlate to AUC/MIC ≥ 600 mg·h/L [4]. Additionally, prolonged therapy exceeding 4 days also heightens the risk, potentially due to the accumulation of the drug in renal tissues [5]. Based on a large meta-analysis authored by Sinha Ray et al., most episodes of AKI developed between 4 and 17 days from the initiation of antibiotic therapy [6].

The nephrotoxic potential of piperacillin–tazobactam, although less extensively studied than that of vancomycin, is also significant, especially when used in combination with other nephrotoxic agents. Studies have indicated that the combination of vancomycin and piperacillin–tazobactam increases the risk of AKI more than either drug alone [7]. Pan and colleagues performed a systematic review and meta-analyses to compare AKI rates with vancomycin combined with piperacillin/tazobactam to vancomycin combined with other anti-pseudomonal beta-lactams, including cefepime or meropenem, and found a significantly higher AKI risk with the vancomycin plus piperacillin/tazobactam combination [8]. The mechanism behind this synergistic nephrotoxicity is not clear yet, but is thought to involve direct tubular toxicity, oxidative stress, and alterations in renal hemodynamics [9].

Patient-specific factors such as age, baseline renal function, and comorbidities can also significantly influence the risk of nephrotoxicity. Elderly patients and those with preexisting renal impairment are particularly vulnerable due to decreased renal clearance leading to increased drug accumulation [3,10]. Furthermore, the presence of comorbid conditions like diabetes mellitus and cardiovascular diseases as well as concomitant use of other nephrotoxic agents, such as nonsteroidal anti-inflammatory drugs (NSAIDs) or aminoglycosides can exacerbate the renal toxicity of these antibiotics [11,12]. In addition, recent studies have highlighted genetic predispositions that may influence susceptibility to drug-induced nephrotoxicity, including polymorphisms in genes encoding drug-metabolizing enzymes and transporters, that can affect the pharmacokinetics of vancomycin and piperacillin–tazobactam, thereby modulating their toxic potential. For instance, variations in the gene encoding the organic anion transporter (OAT) have been linked to differential renal handling of these drugs, impacting their nephrotoxic profiles [13].

The nephrotoxicity associated with vancomycin and piperacillin–tazobactam is a multifaceted issue influenced by drug-specific properties, patient characteristics, concurrent medications, and genetic factors. Understanding these contributing elements at a granular level is essential for optimizing antibiotic use and minimizing the risk of AKI, which remains a significant challenge in clinical practice. For these reasons, we aimed to determine the incidence of AKI in patients receiving vancomycin and piperacillin–tazobactam combination, and to determine potential factors that increased risk of AKI in our patient population.

## 2. Materials and Methods

### 2.1. Study Design and Patients

This is a retrospective, single-center cohort study performed at the Ochsner LSU Health–Academic Medical Center, Shreveport, Louisiana, from May 2019 to March 2023. We included patients ≥ 18 years of age who received at least 48 h of combined intravenous vancomycin and piperacillin–tazobactam. We excluded patients with end-stage renal disease (ESRD) or AKI that developed prior to starting the antibiotics combination regimen as well as patients without a vancomycin serum level collected and/or no repeat serum creatinine (SCr) available. The vancomycin level must have been obtained at steady state, after at least 3 or 4 doses based on maintenance regimen. The primary outcome is the incidence of AKI, with secondary outcomes including the incidence of initial vancomycin trough levels ≥ 20 mg/L, 30-day mortality, and determining independent risk factors contributing to AKI in this population. Factors affecting incidence of AKI will be analyzed, including demographic characteristics, body mass index (BMI), use of concomitant nephrotoxic agents, comorbid conditions, site of infection, use of vasopressors, duration of antibiotic therapy, vancomycin initial trough levels, and estimated AUC/MIC levels. Comorbid conditions that were assessed included hypertension, diabetes mellitus, congestive heart failure, chronic kidney disease, chronic lung disease, cancer, solid organ transplant, human immunodeficiency virus, and urinary obstruction. The use of concomitant nephrotoxic agents was captured, including antibiotics (trimethoprim-sulfamethoxazole, aminoglycosides, colistin), antivirals (acyclovir, foscarnet, tenofovir), antifungals (amphotericin B), NSAIDs, angiotensin-converting enzyme inhibitors (ACEi), diuretics, chemotherapy, immune modulators, intravenous (IV) contrast, and intravenous immunoglobulin (IVIG).

The vancomycin serum level obtained is a trough level drawn between 30 and 60 min prior to the next dose while at steady state. The initial vancomycin trough was drawn as soon as the patient was determined to be at steady state, before the third or fourth dose of vancomycin. If the level was drawn early or late, manual calculations were performed to estimate a correct trough level if drawn correctly. The AUC/MIC value was estimated based on the patient’s trough level, using an online pharmacokinetic calculator with Bayesian modeling, ClinCalc.com/vancomycin [14]. Patients were divided into two groups: acute kidney injury vs. non-acute kidney injury.

### 2.2. Acute Kidney Injury Definitions

AKI definitions were based on two different established criteria: RIFLE (Risk, Injury, Failure, Loss, End-Stage Renal Disease) and AKIN (The Acute Kidney Injury Network).

In RIFLE criteria, R (risk) is identified by a rise in serum creatinine 1.5 times that of baseline, a decrease in glomerular filtration rate (GFR) by 25%, or a urine output < 0.5 mL/kg/h for 6 h. I (injury) is defined by a rise in serum creatinine two times that of baseline, a decrease in GFR by 50%, or a urine output < 0.5 mL/kg/h for 12 h. F (failure) indicates an increase in serum creatinine three times that of baseline, a decrease in GFR by 75%, urine output < 0.3 mL/kg/h for greater than 24 h, or anuria for greater than 12 h. L (loss) is the need for kidney replacement therapy for greater than 4 weeks. E (ESRD) is the need for kidney replacement therapy for greater than 3 months [15].

AKIN criteria are categorized into 3 stages. Stage I is defined by an increase in serum creatinine ≥ 0.3 mg/dL or to 150% to 200% baseline, or urine output < 0.5 mL/kg/h for 6 to 12 h. Stage II is an increase in serum creatinine to 200% to 300% baseline or urine output < 0.5 mL/kg/h for 12 to 24 h. Stage III is an increase in serum creatinine to >300% baseline or increased serum creatinine to ≥4 mg/dL with an acute increase of at least 0.5 mg/dL, urine output < 0.3 mL/kg/h for 24 h or anuria > 12 h, or initiation of kidney replacement [16].

### 2.3. Severity Status Definitions

Sepsis-3 criteria were used to determine the severity status of the patient, including determining sepsis or septic shock. Sepsis is defined as suspected or confirmed infection plus an acute change in the Sequential Organ Failure Assessment (SOFA) score of ≥2 points. Septic shock is defined as a sepsis diagnosis along with persisting hypotension requiring vasopressors to maintain a mean arterial pressure (MAP) ≥ 65 mm Hg and a serum lactate level > 2 mmol/L despite adequate fluid resuscitation [17].

### 2.4. Statistical Analysis

Statistical analysis was performed in SPSS Version 28 (IBM, Armonk, NY, USA). Categorical variables analysis was performed using Pearson chi-square test/Fischer exact test. Continuous variables analysis was performed using Mann–Whitney U tests. All continuous variables are presented as a median with lower and upper quartile ranges. Multivariate logistic regression was run in R version 4.2.2 to assess the factors independently associated with nephrotoxicity. Statistical significance was set at a two-tailed *p*-value < 0.05.

## 3. Results

### 3.1. AKI Rates and Demographics

Out of the total 1153 patients that were screened, 350 patients met the eligibility criteria. Exclusion parameters included: less than 48 h duration of antimicrobials (*n* = 440), missing combination of antimicrobials (*n* = 198), AKI prior to initiation of antimicrobials (*n* = 55), ESRD patients (*n* = 33), missing consecutive doses (*n* = 5), no repeat SCr recorded (*n* = 7), no vancomycin trough level collected (*n* = 50), vancomycin level drawn not at steady state (*n* = 4), oral vancomycin administered (*n* = 3), and pulse dosing (*n* = 8). Among the 350 patients who received a combination of vancomycin and piperacillin/tazobactam, 89 patients (25.4%) developed acute kidney injuries, while 261 patients (74.6%) did not. Details of the AKI staging, as well as baseline SCr immediately prior to combination antimicrobials and maximum SCr while on at least 48 h of combination antimicrobials, are seen in Table 1. There were no statistically significant demographic differences between the AKI and non-AKI groups regarding age, gender, or race, although more AKI cases occurred in African American patients (51.7%). Patients who developed AKI had a higher mean BMI of 31.52 kg/m^2^ ± 13.23 compared to the non-AKI group of 27.35 kg/m^2^ ± 8.11, *p* < 0.001.

Among those with AKI, hypertension was the most common comorbidity (57.3%), followed by diabetes mellitus (23.6%), cancer (20.2%), congestive heart failure (9%), chronic kidney disease (3.4%), chronic liver disease (3.4%), human immunodeficiency virus (1.1%), and urinary obstruction (1.1%). There were no significant differences in the prevalence of these comorbidities between the AKI and non-AKI groups. At least one comorbidity was present in 37.1% of patients in the AKI group compared to 32.6% in the non-AKI group. There was no significant difference in the number of comorbidities per patient between the groups, as seen in Table 2. The 30-day mortality rate was 3.4% in the AKI group compared to 1.95% in the non-AKI group (*p* = 0.42).

### 3.2. Concomitant Agents

We examined various concomitant agents with potential nephrotoxic effects, including antibiotics (trimethoprim–sulfamethoxazole, aminoglycosides, colistin), antivirals (acyclovir, foscarnet, tenofovir), antifungals (amphotericin B), NSAIDs, ACEis, diuretics, chemotherapy, immune modulators, IV contrast, and IVIG. There were no significant differences between the AKI and non-AKI groups for these variables, as seen in Table 3, except for IV contrast use. IV contrast was used in 31.5% of AKI cases compared to 18% in the non-AKI group (*p* = 0.008). Multivariate logistic regression indicated that IV contrast was independently associated with nephrotoxicity (OR = 2.188, 95% CI = 1.026–4.666, *p* = 0.043). No significant difference was observed in the number of concomitant nephrotoxic agents used between the two groups. Although a higher proportion of patients received vasopressors in the AKI group (*n* = 4, 4.5%) compared to the non-AKI group (*n* = 4, 1.5%), the results were not statistically significant (*p* = 0.11).

### 3.3. Sites of Infection

Different sites of infection were observed in both the AKI and non-AKI groups. In the AKI group, the highest proportion was pneumonia at 29.2% (vs. 21.5% in the non-AKI group), while skin and soft tissue infections predominated in the non-AKI group at 31.0% (vs. 21.3% in the AKI group). Other infections included bone and joint infections (23.6% vs. 21.8%), intra-abdominal infections (13.5% vs. 8.8%), bacteremia (4.5% vs. 8.8%), urinary tract infections (2.2% vs. 3.4%), surgical site infections (0 vs. 1.1%), intracranial infections (0 vs. 1.1%), and endocarditis (0 vs. 1.9%). There were no significant differences between the groups in terms of infection sites.

### 3.4. Antimicrobial Administration

In the AKI group, the incidence of an elevated initial vancomycin trough level ≥ 20 mg/L was significantly higher, occurring in 35 patients (39.3%) compared to 30 patients (11.5%) in the non-AKI group, *p* < 0.001. The average duration of dual antimicrobials was longer in the AKI group at 4.65 days ± 2.67 compared to the non-AKI group at 4.04 days ± 1.80, *p* < 0.01. The amount of vancomycin in mg given was higher in the AKI group, which correlates with the higher BMI as vancomycin is dosed according to mg/kg. The median vancomycin loading dose and maintenance doses are summarized in Table 4. The median piperacillin–tazobactam dose given was the same in both groups at 4.5 g. The median initial vancomycin trough level was higher in the AKI group at 17.80 mg/L (IQR, 12.10–24.25) vs. 12.20 mg/L (IQR, 9–16.10), *p* < 0.001, as was the AUC/MIC ratio: 568 mg·h/L (IQR, 427–724) vs. 433 mg·h/L (IQR, 357.50–533.50), *p* < 0.001 between AKI and non-AKI groups. However, multivariate logistic regression analysis revealed no association between the development of AKI and the duration of antibiotics, vancomycin trough levels, or AUC/MIC, as seen in Table 5.

## 4. Discussion

In this study, we found that the AKI rate is similar to those reported in the medical literature, with 25.4% of patients receiving vancomycin/piperacillin–tazobactam combination experiencing an AKI event [18]. Even though the AKIs reported fell under the least severe category according to both RIFLE and AKIN criteria, the impact of any AKI can have heavy consequences. In addition to increased laboratory draws and vancomycin drug monitoring, these AKIs may lead to prolonged hospital stays and difficulty with long-term placement until the AKI resolves.

In our analysis, we demonstrated that patients who developed AKI were significantly more likely to be overweight, with a mean BMI of 31.52 kg/m^2^ compared to 27.35 kg/m^2^ in the non-AKI group (*p* < 0.001). While obesity has previously been associated with increased risk of nephrotoxicity due to altered pharmacokinetics and enhanced drug accumulation in adipose tissue, our vancomycin dosing parameters also reflect higher average doses, as well as higher initial serum levels. This suggests that obesity itself is a marker of increased risk as these patients will receive a larger amount of vancomycin, dosed in mg/kg, and therefore be at increased risk of nephrotoxicity [19].

We showed that the use of concomitant nephrotoxic agents, particularly IV contrast, was significantly higher in the AKI group (31.5% vs. 18.0%, *p* = 0.008). The logistic regression confirmed IV contrast as an independent risk factor for nephrotoxicity (OR = 2.188, 95% CI = 1.026–4.666, *p* = 0.043). This finding underscores the necessity of cautious use of IV contrast in patients receiving nephrotoxic antibiotics. The synergistic nephrotoxicity observed with the combination of vancomycin and piperacillin–tazobactam may be exacerbated by additional nephrotoxic insults from contrast media. This outcome contradicts similar previously published studies examining the impact of concomitant nephrotoxic agents on the risk of AKI when used in combination with vancomycin and piperacillin–tazobactam; therefore, more data is needed on the overall impact of IV contrast use on the development of AKI in such cases [19].

Although our study did not determine an independent association between the development of AKI and the vancomycin trough levels or AUC/MIC, it is important to note the statistically significant differences in levels between the two groups on the univariate analysis. The incidence of initial vancomycin trough levels being supratherapeutic ≥ 20 mg/L was a significant finding in 35 patients (39.3%) in the AKI group compared to 30 patients (11.5%) in the non-AKI group, *p* < 0.001. There are studies which prove that the risk of AKI increases when supratherapeutic levels are reached, specifically those that remain >20 mg/L [1,19]. Although vancomycin dosing is a pharmacist-driven protocol at our institution where pharmacists will adjust the dose and/or frequency if the level is supratherapeutic, initial trough levels being supratherapeutic has been associated with nephrotoxicity in hospitalized patients, and therefore should be avoided if possible [20]. It has been determined that using AUC/MIC dosing results in less use of vancomycin in mg overall, thereby reducing overall drug exposure, and lower rates of AKI [21]. These findings are supported by our study, as the average AUC/MIC in the non-AKI group of 433 mg·h/L is within the efficacy window of 400–600 mg·h/L while avoiding AKI. Our study supports the current guidance from the Infectious Diseases Society of America (IDSA) that suggests moving from trough-based dosing to AUC-based dosing, as AUC-based dosing may provide more patient-specific dosing, especially for obese patients, to decrease the risk of AKI [3,21].

The presence of comorbidities such as hypertension, diabetes mellitus, and chronic kidney disease did not significantly differ between the AKI and non-AKI groups. Recent literature highlights genetic predispositions influencing susceptibility to nephrotoxicity, such as polymorphisms in drug-metabolizing enzymes and transporters, which affect drug pharmacokinetics and dynamics. Although our study did not assess genetic factors, future research incorporating genetic profiling and pharmacogenomics may better elucidate individual susceptibility to drug-induced nephrotoxicity [12].

There are several limitations to this study including being retrospective in nature and not using the KDIGO (Kidney Disease: Improving Global Outcomes) guidelines, which are currently the gold standard for diagnosing AKI. Fluid overload was not assessed, which can have a significant impact on SCr values and documented AKI rates. SCr should ideally be corrected for fluid overload; otherwise, SCr may underestimate AKI severity in this patient population by up to 30% [22]. Other limitations involve information that was not collected in this study, including SCr on admission, blood pressure values, and frequencies of antimicrobial administration. SCr was not collected on admission, which may or may not be more representative of baseline renal function than the SCr prior to administration of antimicrobials. Whereas admission SCr may be falsely elevated due to dehydration, the current method of baseline SCr being collected immediately before administration of antimicrobials may be altered by other factors such as medications or stress from hospital admission. Another parameter not recorded in this study is the blood pressure values in both groups. Even though vasopressor use and septic shock were recorded, a relative fall of blood pressure that was restored with intravenous fluid rather than the use of vasopressor could potentially have an impact on kidney function. In our study, there was no difference in severity status between the two groups of patients; however, limited data was recorded. The frequency of antimicrobial administration was also not specifically documented; however, per our institutional protocol for normal renal function, this indicates a piperacillin/tazobactam dose of 4500 mg every 8 h and vancomycin dosed either every 12 h (most common), every 24 h (for elderly patients or for concerns of impaired clearance), or every 8 h (for young patients with rapid clearance). These regimens and frequencies are changed according to renal function through a pharmacist-driven protocol.

In order to better characterize the impact of IV contrast on the development of AKI, specifics surrounding the use of IV contrast should be studied in more detail. This study did not record the indications for IV contrast, which may have been ordered due to a high severity of infection, and therefore more risk of confounding factors. The time frame of antimicrobial initiation, IV contrast administration, and development of AKI was also not recorded, which may have led to the ability to draw more definitive conclusions regarding which factors had the most impact on renal function. Due to these limitations, this study cannot definitively determine causality regarding the use of IV contrast and AKI; however, we did show an association at our institution.

## 5. Conclusions

At our institution, 25.4% of patients receiving combination therapy of vancomycin and piperacillin–tazobactam have experienced an AKI. Higher BMI and use of concomitant IV contrast were significantly associated with increased risk of nephrotoxicity. Antimicrobial duration, vancomycin trough levels, and estimated AUC/MIC were significantly higher in the AKI groups according to univariate analyses. Therefore, we recommend the judicious use of vancomycin/piperacillin–tazobactam combination in patients with elevated BMI and/or who have received IV contrast. Our study contributes to the body of literature supporting AUC/MIC dosing compared to trough-based dosing to minimize total vancomycin drug exposure and reduce risk of AKI.

Our study underscores the complexity of nephrotoxicity in patients receiving vancomycin and piperacillin–tazobactam. While certain risk factors like BMI and IV contrast use are significantly implicated, the interplay of multiple patient-specific and treatment-related factors necessitates a tailored approach to minimize renal risk. Further research, particularly prospective studies and those incorporating genetic analysis, is essential to refine risk stratification and therapeutic strategies.

## Figures and Tables

**Table 1:** SCr values and AKI descriptions.

SCr Values	AKI *n* = 89 (25.4%)	Non-AKI *n* = 261 (74.6%)
Baseline, Mean (±SD)	0.77 (±0.22)	0.78 (±0.25)
Maximum SCr while on combination therapy	1.56 (±0.78)	0.82 (±0.26)
RIFLE Criteria		
Risk	48 (13.7%)	
Injury	28 (8%)	
Failure	10 (2.9%)	
Loss	3 (0.9%)	
ESRD	0	
AKIN Criteria		
Stage I AKI	60 (17.1%)	
Stage II AKI	19 (5.4%)	
Stage III AKI	10 (2.9%)	

**Table 2:** Baseline characteristics of study population between AKI and non-AKI groups.

Demographics	AKI *n* = 89 (25.4%)	Non-AKI *n* = 261 (74.6%)	*p*-Value
Age, Median (IQR), y	52 (37.50–62)	52 (40–63)	0.18
Gender			
Male	56 (62.9%)	167 (64%)	0.85
BMI, Mean (±SD)	31.52 (±13.23)	27.35 (±8.11)	<0.001
Race			
Black	46 (51.7%)	117 (44.8%)	0.23
White	39 (43.8%)	138 (52.9%)	
Other	4 (4.5%)	6 (2.3%)	
Severity Status			
Sepsis	6 (6.74%)	20 (7.67%)	0.57
Septic Shock	3 (3.37%)	2 (0.77%)	0.68
Intensive Care Unit Care	3 (3.37%)	10 (3.83%)	0.48
Comorbidities			
HTN *	51 (57.3%)	140 (53.6%)	0.54
DM *	21 (23.6%)	71 (27.2%)	0.5
CHF *	8 (9%)	20 (7.7%)	0.69
CKD *	3 (3.4%)	4 (1.5%)	0.28
CLD *	3 (3.4%)	7 (2.7%)	0.73
Cancer	18 (20.2%)	47 (18%)	0.64
SOT *	0 (0.0%)	4 (1.5%)	0.57
HIV *	1 (1.1%)	8 (3.1%)	0.45
Urinary obstruction	1 (1.1%)	3 (1.1%)	1.00
Total number of comorbidities			
1	33 (37.1%)	85 (32.6%)	0.23
2	25 (28.1%)	81 (31.0%)	
3	4 (4.5%)	16 (6.1%)	
4	3 (3.4%)	1 (0.4%)	
5	0 (0.0%)	2 (0.8%)	

* Definitions: HTN, hypertension; DM, diabetes mellitus; CHF, congestive heart failure; CKD, chronic kidney disease; CLD, chronic liver disease; SOT, solid organ transplant; HIV, human immunodeficiency virus.

**Table 3:** Concomitant agents potentially associated with nephrotoxicity.

	AKI, *n* = 89 (25.4%)	Non-AKI, *n* = 261 (74.6%)	*p*-Value
vasopressors	4 (4.5%)	4 (1.5%)	0.11
TMP-SMX *	1 (1.1%)	5 (1.9%)	0.76
Aminoglycosides	3 (3.4%)	6 (2.3%)	
Colistin	0	0	
Acyclovir	1 (1.1%)	3 (1.1%)	0.23
Foscarnet	0	0	
Tenofovir	0	0	
Amphotericin-B	0	0	
NSAIDS *	13 (14.6%)	45 (17.2%)	0.56
ACEi *	19 (21.3%)	48 (18.4%)	0.54
Diuretics	13 (14.6%)	29 (11.1%)	0.38
Chemotherapy	2 (2.2%)	10 (3.8%)	0.73
Immune modulator	1 (1.1%)	6 (2.3%)	0.68
IV contrast	28 (31.5%)	47 (18.0%)	0.008
IVIG *	0 (0.0%)	1 (0.4%)	1.00
Total number of nephrotoxins			
1	33 (37.1%)	100 (38.3%)	0.26
2	19 (21.3%)	39 (14.9%)	
3	3 (3.4%)	9 (3.4%)	
4	1 (1.1%)	0 (0.0%)	

* Definitions: TMP-SMX, trimethoprim–sulfamethoxazole; NSAIDS, nonsteroidal anti-inflammatory drugs; ACEi, angiotensinogen-converting enzyme inhibitors; IVIG, intravenous immune globulin.

**Table 4:** Treatment dosing, levels, and duration parameters.

	AKI	Non-AKI	*p*-Value
	Median (IQR)		
Vancomycin loading dose, mg	1750 (1500–2000)	1500 (1375–2000)	0.009
Vancomycin initial maintenance dose, mg	1250 (1000–1500)	1000 (1000–1250)	0.001
Piperacillin/Tazobactam dose, mg	4500 (3930–4500)	4500 (4500–4.500)	0.86
Vancomycin initial trough at steady state, mg/L	17.80 (12.10–24.25)	12.20 (9–16.10)	<0.001
AUC/MIC, mg·h/L	568 (427–724)	433 (357.50–533.50)	<0.001
Antibiotic duration, days; mean (±SD)	4.65 ± 2.67	4.04 ± 1.80	0.01

**Table 5:** Prediction of risk factors associated with nephrotoxicity.

		95% CI		
Variables	OR	LL	UL	*p*-Value
Age	0.98	0.958	1.002	0.07
Gender	0.99	0.464	2.140	0.99
Race	0.691	0.349	1.370	0.29
BMI	1.027	0.989	1.066	0.163
IV contrast	2.188	1.026	4.666	0.043
Duration of dual antibiotics	1.117	0.962	1.296	0.146
Vancomycin initial trough level ≥ 20 mcg/L	1.564	0.443	5.52	0.487
AUC/MIC ≥ 600 mg·h/L	1.004	0.996	1.012	0.345

## Data Availability

The data presented in this study are available upon request from the corresponding author due to the agreement with the Institutional Review Board to only publicly share information relevant to the study, which is presented in the article.

## Abbreviations

The following abbreviations are used in this manuscript:

AKI	Acute kidney injury
BMI	Body mass index
IV	Intravenous
MRSA	Methicillin-resistant *Staphylococcus aureus*
AUC	Area under the curve
MIC	Minimum inhibitory concentration
NSAIDs	Nonsteroidal anti-inflammatory drugs
OAT	Organic anion transporter
ESRD	End-stage renal disease
ACEi	Angiotensin-converting enzyme inhibitors
IVIG	Intravenous immunoglobulin
RIFLE	Risk, Injury, Failure, Loss, End-Stage Renal Disease
AKIN	Acute Kidney Injury Network
SCr	Serum creatinine
